# Supplementary material for: A set of multi-entry identification keys to African frugivorous flies (Diptera, Tephritidae)
Source: Zookeys. 2014 Jul 24;(428):97–108. doi: 10.3897/zookeys.428.7366 (PMC4143993; doi:10.3897/zookeys.428.7366)
Supplement: Supplementary material 6 — Key to Ceratitis [file zookeys-428-097-s006.zip › SF6_ZooKeys_key to Ceratitis/key/SF6_ZooKeys_key to Ceratitis/Media/Html/Ceratitis whartoni.htm]

Ceratitis dimorphic sp


*Ceratitis (Ceratalaspis) whartoni* De Meyer & Copeland

*Ceratitis whartoni* De Meyer & Copeland
2009: 21.

Body length: 4.8-6.6 mm; wing length: 5.4-6.4 mm.

Male

Head: First flagellomere yellow to orange,
obtuse apically. Arista pubescent, rays at most twice the width of arista at
base. Frontal seta thinner than, and subequal in length to, anterior orbital
seta; two orbital setae, anterior seta longer than posterior one; ocellar seta about
1.5 times as long as ocellar triangle, rarely additional seta of variable
length present but never longer than ocellar; postocellar seta black, shorter
than lateral vertical seta; eye/medial vertical seta ratio (= eye maximum
diameter versus length medial vertical seta): 1.1-1.5. Frons convex; yellow to orange,
with greyish microtrichosity on upper half. Gena broader than in other *Ceratitis*
species, eye/gena ratio (=eye maximum diameter versus height gena) 2.5-3.3; genal
seta and genal setulae yellow, or setulae sometimes blackish.

Thorax: Postpronotal lobe yellowish white;
with black median spot around base of postpronotal seta. Scutum ground colour shining
yellow-brown to dark brown, with silvery microtrichosity covering most of
dorsum except circular area posterior to medial end of transverse suture and in
area extending posteriorly from dorsocentral setae and then narrowly along
sides and demarcated by prescutellar acrostichal, postsutural supra-alar and
intra-alar setae, narrow area along mediolongitudinal line and posterior of postpronotal
lobe with less dense microtrichosity. Anepisternum pale yellow to yellowish
orange, lower third darker; with pale pilosity, one anepisternal seta. Scutellum
yellowish white, with three brownish spots restricted to apical margin and
inferior part of scutellum; partly confluent, connected by yellowish orange
matrix. Subscutellum entirely brown to black. 

Wing: Markings brownish to yellowish
brown. Of typical bands, only anterior apical band distinct; furthermore with brownish
spot (probably fusion of discal and subapical bands) covering area surrounding
cross-vein R-M (i.e. apical margin of cell br) and basal third of cell r4+5,
continued in apical half of cell dm and anterior third of cell m, also broadly
fused with anterior apical band; additional small marks in middle of cell cu_1_
and basal part of cell m. Basal streaks and spots present but poorly
developed. 

Legs: Slender; yellow to yellowish
orange, tarsi sometimes slightly paler than rest of leg; with dispersed pale pilosity
except for fore femur with dark ventral spines, and apical fourth of hind femur
with dark dorsal setae.

Abdomen: Yellow to orange-brown. Abdominal tergites 2 and 4 on posterior
half to two-thirds with greyish microtrichosity. Male genitalia, epandrium
normal shape with well developed surstyli.

Female

As male except for the following
characters: gena less broad, eye/gena ratio 4.1-5; eye/medial vertical seta ratio 1.4-1.7. Wing with well
developed banding as in other *Ceratitis* species: anterior apical band
and discal band touching; subapical band narrowly separated from anterior
apical band, posterior apical band completely isolated. Oviscape orange, with
dispersed dark pilosity. Tergal-oviscapal ratio (= length abdominal tergites 1-5
versus length oviscape): 1.5-2. Aculeus flattened, 7 times longer than broad, apex
bifurcated, and with pair of subapical protuberances.  

(Description after De Meyer &
Copeland, 2009)
